# A Practical Treatise on the Domestic Management and Most Important Diseases of Advanced Life: With an Appendix, Containing a Series of Cases Illustrative of a New and Successful Mode of Treating Lumbago and Other Forms of Chronic Rheumatism, Sciatica, and Other Neuralgic Affections, and Certain Forms of Paralysis

**Published:** 1849-05

**Authors:** 


### REVIEWS.

Art. III.—*A Practical Treatise on the Domestic Management and most important Diseases of Advanced Life:* With an Appendix containing a series of cases illustrative of a new and successful mode of treating Lumbago and other forms of Chronic Rheumatism, Sciatica, and other Neuralgic Affections, and certain forms of Paralysis. By George E. Day, M.D., Fellow of the Royal College of Physicians, and Physician to the Western General Dispensary. 1 vol., 8vo. pp. 226. Philadelphia: Lea & Blanchard. 1839.

"Human life is divisible into three great periods—those of growth, maturity, and decline." In each of these periods there is a difference, to some extent, in the various organs, and in the manner in which they perform their appropriate functions. Of the peculiarities which mark the epochs of growth and maturity we shall not now speak. Let us briefly examine those changes which occur during the progress of decline.

The circulation becomes languid, and hence venous congestions and visceral obstructions, with the various diseases attendant upon them, piles, apoplexy, paralysis, dropsy, gout, diseases of the heart, liver, bladder and kidneys, and chronic affections of the respiratory organs, are more frequent in both sexes: while in the female, diseases of the uterus and mammæ are developed. As age progresses, the energies of the nervous, circulating and muscular systems begin plainly to flag: the digestive functions are more and more impaired; chronic inflammations terminating in organic changes are oftener observed; the force of vital resistance is diminished, and hence we so often see senile gangrene: appoplexy and paralysis; comatose affections resulting from diminished nervous energy; disorders of the urinary apparatus in the male; and passive hemorrhages, marking a want of tone in the system are now more frequent.

As the individual grows older, the whole physical and mental powers rapidly decline. The sunken cheek, the pallid face, the dry and wrinkled skin, the feeble limbs, the tottering gait, the bended trunk, the tremulous tongue, and the

> "Big manly voice
> Turning again toward childish treble, pipes
> And whistles in his sound,"

proclaim the decay of the body, while the lacklustre eye, the expressionless features, the feeble manifestations of intellect, the failing memory, all bear witness that the

> "Last scene of all
> That ends this strange eventful history,
> Is second childishness and mere oblivion."

It may not be out of place to examine here some of the changes occurring in advanced life; for there certainly are at this time, in the most essential organs, peculiarities modifying greatly the due performance of their functions, and influencing the ordinary progress of disease and its proper treatment. Our author considers at some length "the alterations in structure and function of the organs most liable to be affected in old age." Let us briefly and succinctly notice some of these: and

1. Of the *respiratory organs* and their functions. The walls of the chest are altered in form—the transverse and vertical diameters are diminished, and there is anterior curvature of the spine. The intercostal spaces are lessened and occasionally even the ribs unite—the cartilages of the first two are commonly ossified. The form of the diaphragm is changed; nor do the lungs remain unaffected. The position and direction of the fissures are altered: the air cells are enlarged; crepitation on pressure is no longer heard; the organs themselves are smaller than in the previous periods of life. They may be livid and very flabby, so that their natural conical form is lost, while they are incapable of being much increased in size by inflation and contain only a very small portion of that fine vascular tissue so perceptible in the adult lung. A black deposite is often found in them, due, perhaps, to the stagnation of blood in the pulmonary tissues, and producing, when abundant, obstructions to the action of the capillaries and bronchial tubes—which latter are also lessened in size by hypertrophy of their fibrous structure, chalky deposits in the submucous tissue, &c. The absorbents are diminished. The thyroid and cricoid cartilages as well as the rings of the trachea and bronchi are often ossified.

These changes in the structure of these organs necessarily modify the performance of their functions. Hence we see that the movements of the chest are less extensive and more irregular; the diaphragm is almost the exclusive agent in respiration; the diminished size and expansive power of the lungs and the decrease of their vascular tissue no longer allow the free aeration of the blood; less oxygen is taken in and less carbon thrown off. The quantity of aqueous vapor charged with organic matter commonly evolved from the lungs, is now less in amount because of the altered condition of the vascular tissue and mucous membrane—and hence the lungs have a tendency to become edematous.

2. Modifications in the nervous system and its functions. While the skull is considerably diminished in thickness, the dura mater adheres closely to the bone; the arachnoid and pia mater become thickened, tough and rather opaque, and not unfrequently calcareous deposits, or vesicles filled with fluid are found on the surface of the latter; there is usually a considerable quantity of fluid in the sub-arachnoid space and the ventricles are nearly always more or less distended by an effusion varying in quantity and appearance. It has been suggested (first we believe, by Dr. Todd, *Cyclopedia of Anatomy and Physiology, vol.* 3,) that this increased amount of fluid within and around the nervous center, though it be the result of a change which depresses the general nutrition of the organ, is yet *conservative* in its influence. The brain is smaller in size; the convolutions are shrunken and the sulci between them increased in width, while the external layer of gray matter is decidedly thinner than in the period of maturity. Its quantity of blood is diminished and the vessels become thickened and lose their contractile powers.

The special senses, so intimately connected with the nervous system, also undergo many changes.

The fluids of the eye are lessened in quantity; the cornea becomes flattened—the *arcus senilis* makes its appearance—the iris and choroid coat turn paler and the pupils grow smaller; the lens flattens; the vitrous humor is less transparent; the lachrymal puncta frequently close—when there will be a constant flow of tears.

All the structures of the ear are hardened; the bones unite; the tympanum becomes tense; the fluid of the internal ear is lessened in amount and the auditory nerve is harder and smaller thsn in the adult.

The tongue is altered. The Schneiderian membrane is dry and secretes but little mucus. The epidermis is rough, dry and withered.

The functional changes are equally well marked. The senses become blunted; the perceptions are less keen; the energy of volition is enfeebled; and irritability and sensibility are much diminished. Sympathy is greatly impaired, so that the symptoms of a given disease are more strictly confined to the suffering organ and are oftener masked and obscure. "A lung," for example, says our author, "may be perfectly impermeable or may even be disorganized, yet the heart may afford no indication either by its force or its frequency that one of the most essential of the vital functions is fast ceasing."

3. The *digestive organs* and their functions. The mucus membrane of the stomach and intestines is much thickened, and thus diminishes their capacity; the muscular coat is often completely atrophied; the villi and mucus follicles are shrunken, and but little mucus is secreted. The imperfect mastication and the admixture of saliva, gastric juice and bile, modified doubtless by age, added to this condition of the stomach will abundantly explain the diminished nutrition of old age. The dyspepsia of this period of life is owing in part to these causes, and in part also to the diminished, nervous energy.

4. *Of the organs of circulation.* The heart generally diminishes, but occasionally owing to the resistence offered to the prompt escape of the blood, increases in size. The free margins of the valves are thickened; deposits of calcareous matter are found in the arteries; sometimes they are completely ossified; the walls of the capillaries are thickened, hindering the free passage of the blood from the arteries to the veins, and in this way overcoming in a large measure the propelling force of the heart and arteries, gives rise to congestions and brings about a tortuous and varicose condition of the veins.

The action of the heart may be weak—when dropsical effusions and a general failure of all the functions of the body will result—or inordinately strong, when the smaller blood-vessels of the brain particularly, are apt to be ruptured and thus produce appoplexy and paralysis.

An important practical deduction is drawn from the diseased condition of the arteries in old age, viz:—that the *pulse should always be counted at the heart.*

The alteration of structure and function in the *urinary apparatus*, we will not now notice. We have already exceeded our limits.

Such organic changes cannot fail to modify disease and influence materially the treatment. It is the object of the author of the volume before us, to discuss both these points. A work of this kind was certainly needed, for with the single exception of Canstatt, no writer, so far as we now recollect, has given to the world a systematic work on "the *diseases of old age.*" A multitude of original and valuable monographs on individual points connected with this general subject have appeared at different periods, but even they are "scattered among the French and German periodicals" and consequently inaccessible to most English readers.

"I wish it to be distinctly understood, "says the author, "that it has not been my object to write comprehensive essays on these various diseases. All that I have attempted to do, has been to explain the modifications that advanced life impresses on the different symptoms, and to point out the peculiarities in the mode of treatmemt that should be adopted during the declining period of the vital power."

This task has been judiciously executed, and we trust the work will have a circulation commensurate with the importance of the subject and the creditable manner in which it has been completed.

The Appendix contains a "series of cases illustrative of a new and successful mode of treating Lumbago," &c.

This method "consists essentially in the instantaneous application of a flat iron button, gently heated in a spirit lamp, to the skin." This button is about half an inch in diameter and a quarter in thickness, and is connected with a small wooden handle by an iron shank curved nearly at a right angle, at about half an inch from the upper surface of the button.

The button is held in the flame of a spirit lamp for a few seconds (until the curved portion of the shank feels uncomfortably warm to the fingers) and is then rapidly and lightly drawn over the affected part. When properly performed, this operation never vesicates, but produces only a slight redness.

The following cases are selected as fair specimens from a number detailed in this part of the work.

"CASE I.—J. A., aged forty, an omnibus conductor, of healthy appearance, and good muscular development. Had frequently suffered from lumbago for several years past. Caught a severe cold in February, but continued his ordinary avocation for some days, till one morning, on attempting to rise, the pain was so severe, that, to use his own expression, he 'was obliged to hollow out.' He then became a patient at the Western General Dispensary, and remained for upwards of two months under the care of one of my colleagues, who found all the ordinary treatment unavailing in removing the lumbar pains, and knowing that I was engaged in investigating the therapeutic value of the thermic treatment, kindly consented to his being transferred to me. After two applications of the iron at intervals of two days, he said he felt himself fit to resume his work. I met him about a month afterwards, when he told me he had felt no return of the pain since he had previously seen me."

"CASE X.*—'Early in the month of May, 1845, I was rather suddenly attacked with severe pains in the right thigh, extending from the hip to the knee. Having never suffered from any similar affection, and generally having good health, I hoped this pain would have yielded to rest, and the warm bath, which last remedy I had recourse to after a week's suffering. This was not the case, I only obtained temporary relief; the pain now became more fixed, and was so increased in intensity, that I could not walk the shortest distance without being obliged to stand at every twenty or thirty paces to obtain ease,

* The patient in this case was a medical man and tells his own story.

which I did the moment I stopped. The entire thigh participated in the affection, but I felt the pain principally along the course of the sciatic and anterior crural nerves; I could not endure to lie on the affected side, as doing so considerably increased the pain. I continued to suffer in this manner for a period of six months, during which time I had recourse to the usual remedies of warm baths, frictions, strong stimulating liniments, hyd. potassæ and morphia in combination, tinct. aconit., electro-magnetism, and all without any effect more than temporary relief. Changes of temperature produced very little, if any effect. I then applied to Dr. Corrigan, who immediately applied the hot iron, from which application I received considerable relief, so much so, that in two days I was more free from pain than I had been from the entire period since the commeneement of the attack. The first day that the last remedy was tried, I had a distance of about half a mile to walk to Dr. Corrigan's house, which occupied me nearly an hour, being obliged to stand at least fifty times to obtain relief. I did not go to Dr. Corrigan until after a week, and on my second visit I was able to walk the same distance without once resting. After about eight applications of the hot iron in the space of four or five weeks, I became perfectly free from pain, now being able to walk a considerable distance without much inconvenience, I discontinued my attendance on Dr. Corrigan. Several weeks passed without my being at all troubled by any return of the pain; but within the last fortnight I have had some temporary attacks. I think it right to say I feel satisfied that if I had continued the application of this remedy some time longer, the cure would have been complete.'

"I trust that the cases I have given will be regarded as furnishing sufficient evidence of the singular efficacy of the thermic treatment in a class of diseases which are usually regarded as of a most intractable and obstinate character. I may add that I have successfully applied it in spinal irritation, in various painful manifestations of hysteria, and in many forms of neuralgia; and I cannot conclude without again expressing my conviction that the practitioner who will give the thermic treatment a fair trial, will not readily abandon a remedial agent by which human suffering can be so easily and rapidly alleviated."

This certainly is success as unusual as it is flattering. This mode of treatment was first introduced to the notice of the profession by the late Sir A. Carlisle, some twenty-five years ago, but has never been used to a very great extent.

Whether or not further experience will confirm the high estimate set by Drs. Day and Corrigan upon this "Thermic treatment," as it is called, of these forms of disease, of course cannot now be be told. The matter is worthy of a more extended trial. R. J. B.

